# Thymic stromal lymphopoietin is a key cytokine for the immunomodulation of atherogenesis with Freund's adjuvant

**DOI:** 10.1111/jcmm.15235

**Published:** 2020-04-13

**Authors:** Martin Steinmetz, Ludivine Laurans, Sarah Nordsiek, Lena Weiß, Bieke van der Veken, Padmapriya Ponnuswamy, Bruno Esposito, Marie Vandestienne, Andreas Giraud, Cristina Göbbel, Eva Steffen, Tobias Radecke, Stephane Potteaux, Georg Nickenig, Tienush Rassaf, Alain Tedgui, Ziad Mallat

**Affiliations:** ^1^ Klinik für Kardiologie und Angiologie Westdeutsches Herz‐ und Gefäßzentrum Universitätsklinikum Essen Essen Germany; ^2^ Paris Cardiovascular Research Center INSERM U970 Paris France; ^3^ Medizinische Klinik und Poliklinik II Universitätsklinikum Bonn Bonn Germany; ^4^ Division of Cardiovascular Medicine Addenbrooke's Hospital University of Cambridge Cambridge UK

**Keywords:** apolipoprotein E, atherosclerosis, Freund's adjuvant, immunization, lymphocytes, monocytes, thymic stromal lymphopoietin

## Abstract

Adaptive immune responses regulate the development of atherosclerosis, with a detrimental effect of type 1 but a protective role of type 2 immune responses. Immunization of Apolipoprotein E‐deficient (ApoE^−/−^) mice with Freund's adjuvant inhibits the development of atherosclerosis. However, the underlying mechanisms are not fully understood. Thymic stromal lymphopoietin (TSLP) is an IL7‐like cytokine with essential impact on type 2 immune responses (Th2). Thymic stromal lymphopoietin is strongly expressed in epithelial cells of the skin, but also in various immune cells following appropriate stimulation. In this study, we investigated whether TSLP may be crucial for the anti‐atherogenic effect of Freund's adjuvant. Subcutaneous injection of complete Freund's adjuvant (CFA) rapidly led to the expression of TSLP and IL1*β* at the site of injection. In male mice, CFA‐induced TSLP occurred in immigrated monocytes—and not epithelial cells—and was dependent on NLRP3 inflammasome activation and IL1*β*‐signalling. In females, CFA‐induced TSLP was independent of IL1*β* and upon ovariectomy. CFA/OVA led to a more pronounced imbalance of the T cell response in TSLPR^−/−^ mice, with increased INF*γ*/IL4 ratio compared with wild‐type controls. To test whether TSLP contributes to the anti‐atherogenic effects of Freund's adjuvant, we treated ApoE^−/−^ and ApoE^−/−^/TSLPR^−/−^ mice with either CFA/IFA or PBS. ApoE^−/−^ mice showed less atherogenesis upon CFA/IFA compared with PBS injections. ApoE^−/−^/TSLPR^−/−^ mice had no attenuation of atherogenesis upon CFA/IFA treatment. Freund's adjuvant executes significant immune‐modulating effects via TSLP induction. TSLP‐TSLPR signalling is critical for CFA/IFA‐mediated attenuation of atherosclerosis.

## 1 INTRODUCTION

Atherosclerosis is a chronic inflammatory disease.^1^ It is characterized by the complex immunological interaction of residential vascular cells and professional immunocytes. Monocytes enter the lesion area and differentiate into dendritic cells or macrophages that incorporate, process, present and are activated by oxidized low‐density lipoproteins (oxLDL) and other antigens. Whereas classical monocytes may be responsible only for the perpetuated pro‐inflammatory response, non‐classical monocytes and type 2 macrophages have also been linked to repair and reconstitution at the site of inflammation.^2^ T cells are activated in peripheral lymphoid organs but also adhere to and migrate into arterial lesions. CD4‐positive T cells react to antigen presentation by dendritic cells and in return, foster the activation of immune (eg macrophages) and vascular cells, mainly through cytokine production. T helper (Th) type 1 cells mostly produce interferon (IFN)‐γ and TNF and promote atherogenesis, whereas Th2 cells mostly produce type 2 cytokines IL(interleukin)4, IL5 and IL13, which are mainly associated with anti‐atherogenic properties.^3^

Manipulation of these immunological interactions may have important therapeutic consequences as suggested by various studies^4^ like recently CANTOS (Canakinumab Anti‐inflammatory Thrombosis Outcomes Study), in which the alteration of chronic inflammation with the anti‐IL1β antibody Canakinumab reduced atherosclerosis independently and was introduced as an alternative therapy beyond lipid lowering.^5^ Immunization of Apolipoprotein E‐deficient (ApoE^−/−^) mice with immuno‐adjuvants in combination with low‐density lipoprotein (LDL)‐derived antigens reduces the progression of atherosclerotic lesions.^6^ Interestingly, the repetitive administration of the water‐oil–based Freund's adjuvant without additional antigens can also slow down atherogenesis, though to a lesser degree than the adjuvant/antigen regimen.^6^, ^7^ The underlying mechanisms have not been fully understood. Interestingly, however, the anti‐atherogenic effect of Freund's adjuvant is lost in mice depleted of CD4 T cells.^6^ We hypothesized that local processes at the site of injection may be pivotal in the induction of the atheroprotective immune response.

Thymic stromal lymphopoietin (TSLP) is an IL7‐like cytokine that is crucial for the induction of type 2 immunity.^8^ Its cellular sources are mainly epithelial cells in the skin (eg keratinocytes), but also murine plasmacytoid and especially myeloid dendritic cells, or human monocyte‐derived dendritic cells.^9^ The TSLP receptor consists of IL7Rα and a TSLP‐specific subunit and is expressed on innate and adaptive immune cells. Recent studies confirm its role as a master regulator for Th2 inflammatory responses in allergic diseases. In B lymphocytes, TSLP promotes their maturation to the B220/IgM stage; in T cells, it directly acts on proliferation and survival and can directly induce IL4 production in naïve CD4 T cells.^10^, ^11^

In this study, we investigated, whether TSLP is crucial for the reduction of atherosclerosis by Freund's adjuvant.

## 2 METHODS

### 2.1 Mice

C57BL/6 wild‐type mice were purchased from Janvier Labs. TSLPR‐deficient (TSLPR^−/−^) mice were kindly provided by Dr Christophe Heymes and originally from Jax labs. Apolipoprotein E‐deficient (ApoE^−/−^) and RAG1‐deficient (RAG1^−/−^) mice are originally from the Jax labs. IL1β‐deficient (IL1β^−/−^) mice were originally kindly provided by Prof. Suda. NLRP3‐deficient (NLRP3^−/−^) mice were bought from Dr Bernhard Ryffel. ApoE^−/−^/TSLPR^−/−^ mice were generated by crossing both lines. CCR2‐deficient (CCR2^−/−^) mice were kindly provided by Dr Christophe Combadiere.

Mice received standard chow and water ad libitum. Experiments were conducted according to the French veterinary guidelines and those formulated by the European Community for experimental animal use (L358‐86/609EEC) and were approved by the Institut National de la Santé et de la Recherche Médicale.

### 2.2 Treatment regimen

For immunization experiments (Table S1), the mice were injected a total volume of 200 µL subcutaneously: Freund's adjuvant (complete or incomplete, both Sigma) and Alum (Imject™ Alum Adjuvant, Thermo Fisher Scientific) were prepared 1:1 with phosphate buffered saline (PBS). ODN 1668 (Sigma) was used as oligodeoxynucleotide and dissolved in PBS. Antigens were solved in PBS at the indicated concentrations. Chicken ovalbumin (Sigma) was emulsified in CFA and administered subcutaneously at a concentration of 100 µg per mouse. For later PCR analysis, the skin area of injection was explanted after killing and immediately shock frozen in liquid nitrogen. Samples for immunohistochemistry were directly frozen in Tissue‐Tek OCT compound (Sakura) at −20°C for 1 hour and then transferred to −80°C.

Circulating monocytes were depleted using liposomes containing clodronate (dichloromethylene diphosphonate) (ClodronateLiposomes.org). Animals received 150 μL intravenous injections of clodronate‐ or PBS‐liposomes. Depletion was verified by flow cytometry.

### 2.3 RNA isolation and reverse transcription

For isolation of RNA from mouse skin, explanted parts (approx. 0.5 × 0.5 × 0.5 cm^3^) were placed in 1 mL TRIzol Reagent (Thermo Fisher Scientific) and disrupted with a Polytron homogenizer (Thomas Scientific). Debris was removed through centrifugation for 10 minutes at 7500 g. The supernatant was transferred and then further processed according to the manufacturer's protocol. The RNA pellet was finally dissolved in 100 µL RNAse/DNAse free water and quantified with a NanoDrop. cDNA synthesis was performed with QuantiTect Reverse Transcription Kit (Qiagen).

### 2.4 Quantitative real‐time PCR

Quantitative real‐time PCR (polymerase chain reaction) was performed on a StepOnePlus Real‐Time PCR System (Applied Biosystems) using SYBR green PCR master mix (Applied Biosystems). Cycle thresholds for **GAPDH** (primers: F, 5′‐GCC GTG AGT GGA GTC ATA CTG GAA CA‐3′; R 5′ – CGT CCC GTA GAC AAA ATG GTG AA‐3′) or **β‐actin** (F 5′ – CAC TGT GCC CAT CTA CGA – 3′; R 5′ – GTA GTC TGT CAG GTC CCG – 3′) were used to normalize gene expression. The following primer sequences were used:

**IL1β**: F 5′ – GAA GAG CCC ATC CTC TGT GA – 3′, R 5′ – GGG TGT GCC GTC TTT CAT TA – 3′; **TSLP**: F 5′ – TGA CTG GAG ATT TGA AAG GGG CTA AGT – 3′; R 5′ – CTT GTT CTC CGG GCA AAT GTT TTG T – 3′; **IL‐6**: F 5′ – AAA GAC AAA GCC AGA GTC CTT CAG AGA GAT – 3′, R 5′ – GGT CTT GGT CCT TAG CCA CTC CTT CTG T – 3′; **TNFα**: F 5′ – GAT GGG GGG CTT CCA GAA CT – 3′, R 5′ – CGT GGG CTA CAG GCT TGT CAC – 3′; **INFβ**: F 5′ – CCA CCA CAG CCC TCT CCA TCA ACT AT – 3′, R 5′ – GGA TCT TGA AGT CCG CCC TGT AGG T – 3′; **INFγ**: F 5′ – AGG AAC TGG CAA AAG GAT GGT GA – 3′; R 5′ – CGC TGG ACC TGT GGG TTG T – 3′.

### 2.5 Flow cytometry

Peripheral blood samples were used for the detection of monocyte subsets upon clodronate treatment. For the analysis of TSLP expression in the skin, excised tissue samples were placed into a cocktail of collagenase I, collagenase XI, DNase I and hyaluronidase (Sigma‐Aldrich); and shaken at 37°C for 1 hour. Cells were then triturated and centrifuged (15 minutes, 500 g, 4°C).

Cells were labelled with anti – CD11b – FITC or eFluor 450 (eBioscience), anti–Gr1‐PerCP‐Cy5.5 (eBioscience), anti‐CD115‐PE (eBioscience), anti–CD11c‐PE‐Cy7 (eBioscience) or anti–CD45‐FITC (eBioscience) and then analysed by flow cytometry on a Fortessa cytometer (Becton Dickinson). For intracellular cytokine staining, surface staining was performed before permeabilization using an intracellular staining kit (eBioscience). TSLP was labelled with anti‐TSLP‐Alexa Fluor 488 (Bioss Antibodies).

### 2.6 Histology

Skin samples were directly frozen in OCT at −20° for 1 hour and then transferred to −80°C. Transversal 10 µm sections were thawed and fixed with acetone at 4°C for 10 minutes before further staining according to standard protocols. Briefly, the samples were blocked with 10% FCS for 30 minutes and then incubated with the primary antibody (anti‐TSLP, Santa Cruz or anti‐MOMA2, Acris) for 120 minutes at room temperature. After washing with PBS, they were incubated with a secondary antibody for 60 minutes. Nuclei were counterstained with DAPI. Hearts were stored in 4% paraformaldehyde at 4°C. Before inclusion in a cutting medium and further storage at −80°C, hearts were treated with 10% sucrose in phosphate buffered saline for 24 hours. Successive 10 µm transversal sections of aortic sinus were obtained. Lipids were detected using Oil Red O staining, monocytes/macrophages with anti‐MOMA2 (Acris).

### 2.7 ELISpot and ELISA

A total of 500 000 spleen cells per well were used, and all samples were used in triplicates. Cells were cultured in RPMI 1640 supplemented with Glutamax, 10% FCS, 0.02 mmol/L *β*‐mercaptoethanol and antibiotics Penicillin and Streptomycin (referred to as “complete” RPMI). ELISpot assays for IFNγ (BD) and IL4 (BD) were performed according to manufacturer's instructions. Cells were incubated for 24 hours (IFNγ) or 48 hours (IL4, IL17) in complete RPMI in the presence of 100 µg/mL chicken ovalbumin (Sigma) as indicated. For immunoglobulin measurement, a standard ELISA was performed with chicken Ovalbumin coating (1 mg/mL PBS) and goat anti‐mouse IgG1 (SouthernBiotech) or goat anti‐mouse IgG2c (SoutherBiotech).

### 2.8 Statistical analysis

Data are expressed as mean ± SEM. Data were compared, and intergroup differences were analysed with one‐way ANOVA and post hoc Tukey's test. Other data were analysed by a two‐tailed Student's *t* test. Differences were considered statistically significant when the probability value was ≤.05.

## 3 RESULTS

### 3.1 Freund's adjuvant induces the expression of TSLP at injection site in C57bl/6J wild‐type and ApoE^−/−^ mice

**FIGURE 1:**
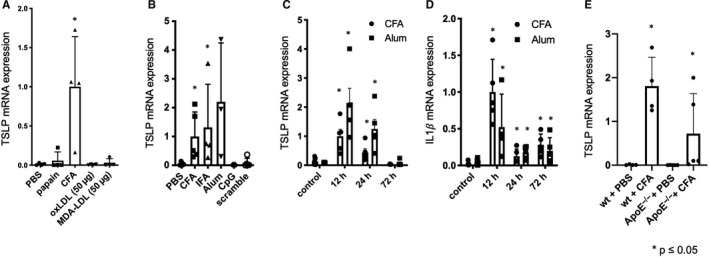
Adjuvants induce TSLP at the site of injection. A, TSLP mRNA expression (relative to GAPDH) upon s.c. application of papain, CFA, oxLDL and MDA‐LDL. B, TSLP mRNA expression (relative to GAPDH) upon application of CFA, IFA, Alum, CpG and scramble control. C, TSLP mRNA expression and D, IL1*β* mRNA expression (relative to GAPDH) in a kinetic analysis 12 h, 24 h and 72 h after the s.c. injection of CFA or Alum. E, TSLP mRNA expression (relative to GAPDH) in wild‐type and ApoE^−/−^ mice upon s.c. CFA injection. *P ≤ .05, n = 3‐5. s.c., subcutaneous; PBS, phosphate buffered saline; CFA, complete Freund's adjuvant; IFA, incomplete Freund's adjuvant; oxLDL, oxidized low‐density lipoprotein; MDA‐LDL, malondialdehyde‐modified low‐density lipoprotein; ApoE, Apolipoprotein E

Atherosclerosis can be modified by the administration of selected immuno‐adjuvants in combination with specific antigens, but also if only adjuvants are applied. Hence, we initially investigated whether TSLP is expressed upon the injection of adjuvants and/or immunogenic antigens in wild‐type mice. Tang et al suggested that the adjuvant papain may induce TSLP mRNA in mouse ears at the site of injection peaking 12 hours after injection.^12^ We did not find a significant increase of TSLP upon papain expression 12 hours after subcutaneous injection in the back skin. However, equal volumes of complete Freund's adjuvant‐induced TSLP, whereas the immunogenic antigens oxidized LDL (oxLDL) or malondialdehyde‐modified LDL (MDA‐LDL) did not (Figure 1A). Thus, we compared several immuno‐adjuvants with known impact on atherogenesis. Freund's adjuvant (complete and incomplete) and Alum, previously shown to limit atherogenesis,^6^, ^7^ likewise induced TSLP, but CpG 1668 – oligodeoxynucleotides, used as an adjuvant for vaccination, which promotes atherogenesis,^13^ had no effect on TSLP expression (Figure 1B). Among possible TSLP‐inducing cytokines, only IL1β was significantly induced at the injection site in response to CFA (Figures S1 and S2). Further, a kinetic analysis revealed a peak at 12 hours for both TSLP (Figure 1C) and IL1β (Figure 1D). We applied the same model to ApoE^−/−^ mice and found that TSLP was also strongly induced in their skin 12 hours upon s.c. injection of CFA (Figure 1E).

### 3.2 Inflammasome‐dependent IL1β signalling induces TSLP in the skin

**FIGURE 2:**
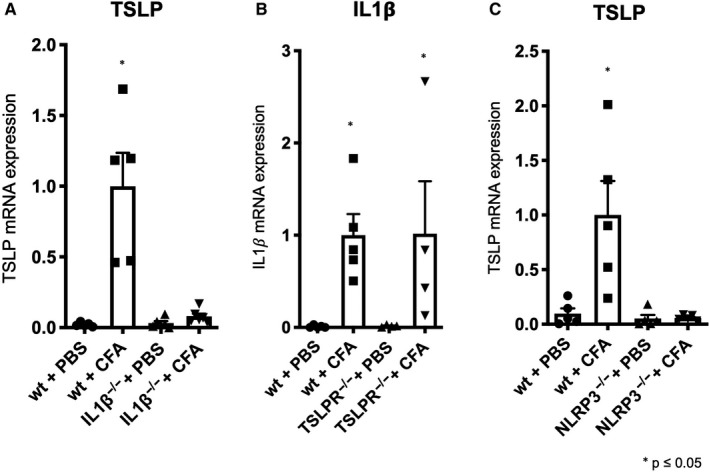
TSLP is induced by inflammasome‐dependent IL1*β* signalling. A, TSLP mRNA expression (relative to GAPDH) upon s.c. application of CFA in IL1*β* ^−/−^ mice and wild‐type mice 12 h after injection. B, TSLP mRNA expression (relative to GAPDH) upon s.c. application of CFA in TSLPR^−/−^ wild‐type mice 12 h after injection. C, TSLP mRNA expression (relative to GAPDH) upon s.c. application of CFA in NRLP3^−/−^ mice 12 h after injection. *P ≤ .05, n = 4‐5. PBS, phosphate buffered saline; CFA, complete Freund's adjuvant; IL1*β*, interleukin‐1*β*; TLSPR, thymic stromal lymphopoietin receptor; NLRP3, Nacht, LRR and PYD domains‐containing protein 3

IL1β and TSLP were both induced upon CFA injection and peaked at 12 hours. When analysed at a much earlier time‐point (4 hours after injection), IL1β was mildly, but statistically significantly increased upon CFA, whereas TSLP was not (Figure S3). In order to understand whether those cytokines mutually influenced each other, we tested CFA in IL1β^−/−^ ‐ or TSLPR^−/−^ mice. IL1β deficiency abolished CFA‐dependent TSLP induction (Figure 2A). Interestingly, we found that this effect was strictly gender‐dependent, because female IL1β^−/−^ mice still expressed TSLP unless they were ovariectomized (Figure S4A‐C). TSLPR deficiency had no impact on IL1β induction by CFA (Figure 2B).

Next, we sought to evaluate the pathway upstream of IL1β expression. CFA‐induced TSLP expression was abrogated in NLRP3^−/−^ mice, upstream of IL1β production (Figure 2C). Thus, NLRP3 inflammasome‐dependent IL1β generation is essential for TSLP expression in response to CFA.

### 3.3 Monocytes significantly contribute to TSLP expression at injection site

**FIGURE 3:**
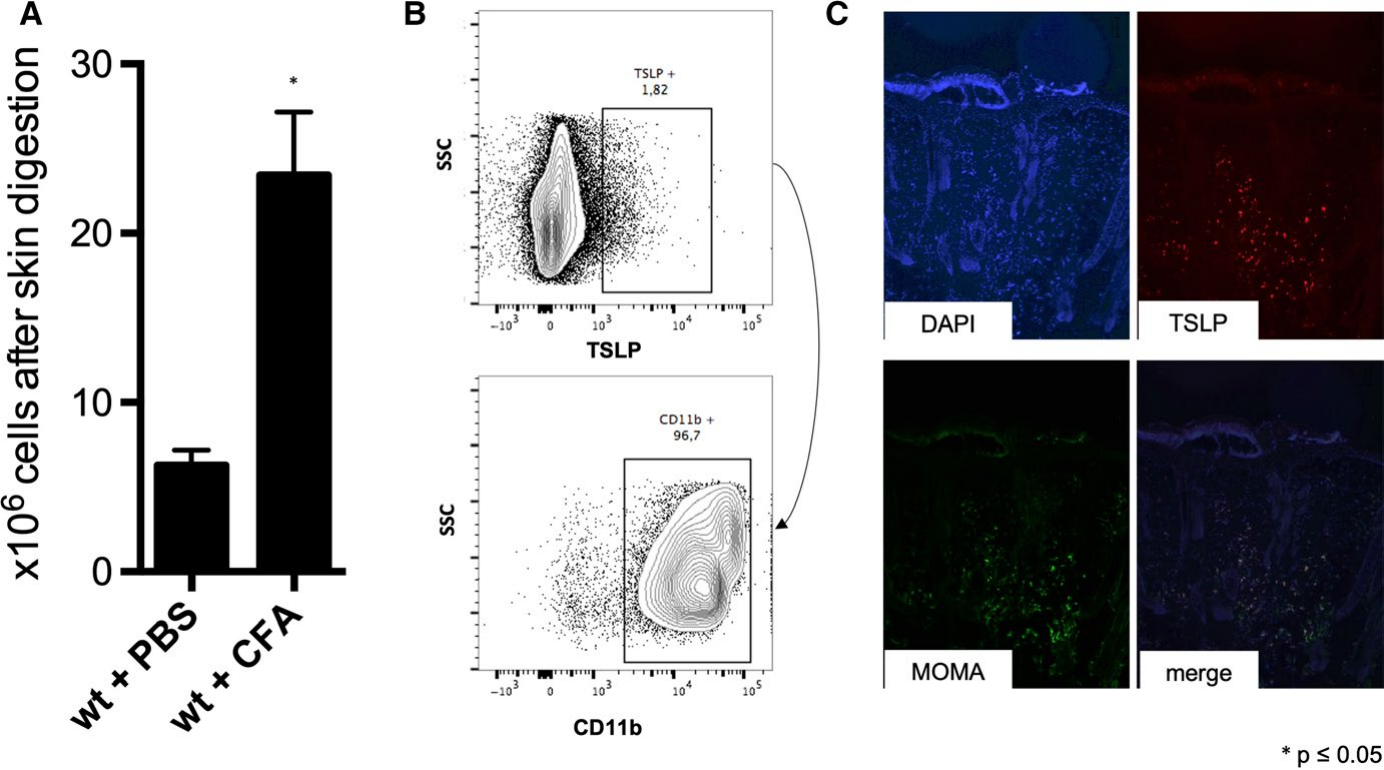
TSLP‐positive monocytes and macrophages at the site of injection. A, Quantification of cell infiltrate in the injected skin area after extraction and digestion of the skin sample with a cell counter. B, Flow cytometry of cellular infiltrate in the injected skin area. C, Histological samples of TSLP‐positive and MOMA‐positive cells in the injected skin area. *P ≤ .05, n = 3. PBS, phosphate buffered saline; CFA, complete Freund's adjuvant; DAPI, 4′,6‐diamidino‐2‐phenylindole; MOMA, anti‐monocyte and macrophage antibody

T and B lymphocytes appear not to contribute to TSLP expression, since RAG1^−/−^ mice still feature a significant TSLP induction upon CFA injection (Figure S5). To further identify the cellular subsets that are responsible for IL1β and TSLP expression, we explanted the local skin 12 hours after injection, digested the specimen and stained for CD45, TSLP, CD11b and CD11c. The CFA‐injected skin featured a significantly higher number of cells (Figure 3A). Among the CD45/TSLP‐positive cells, the majority were positive for CD11b (Figure 3B, Figure S6). Finally, in an immuno‐histological analysis of explanted skin specimens that were injected with CFA, TSLP was co‐expressed in MOMA‐positive cells (which are monocytes/macrophages) (Figure 3C).

**FIGURE 4:**
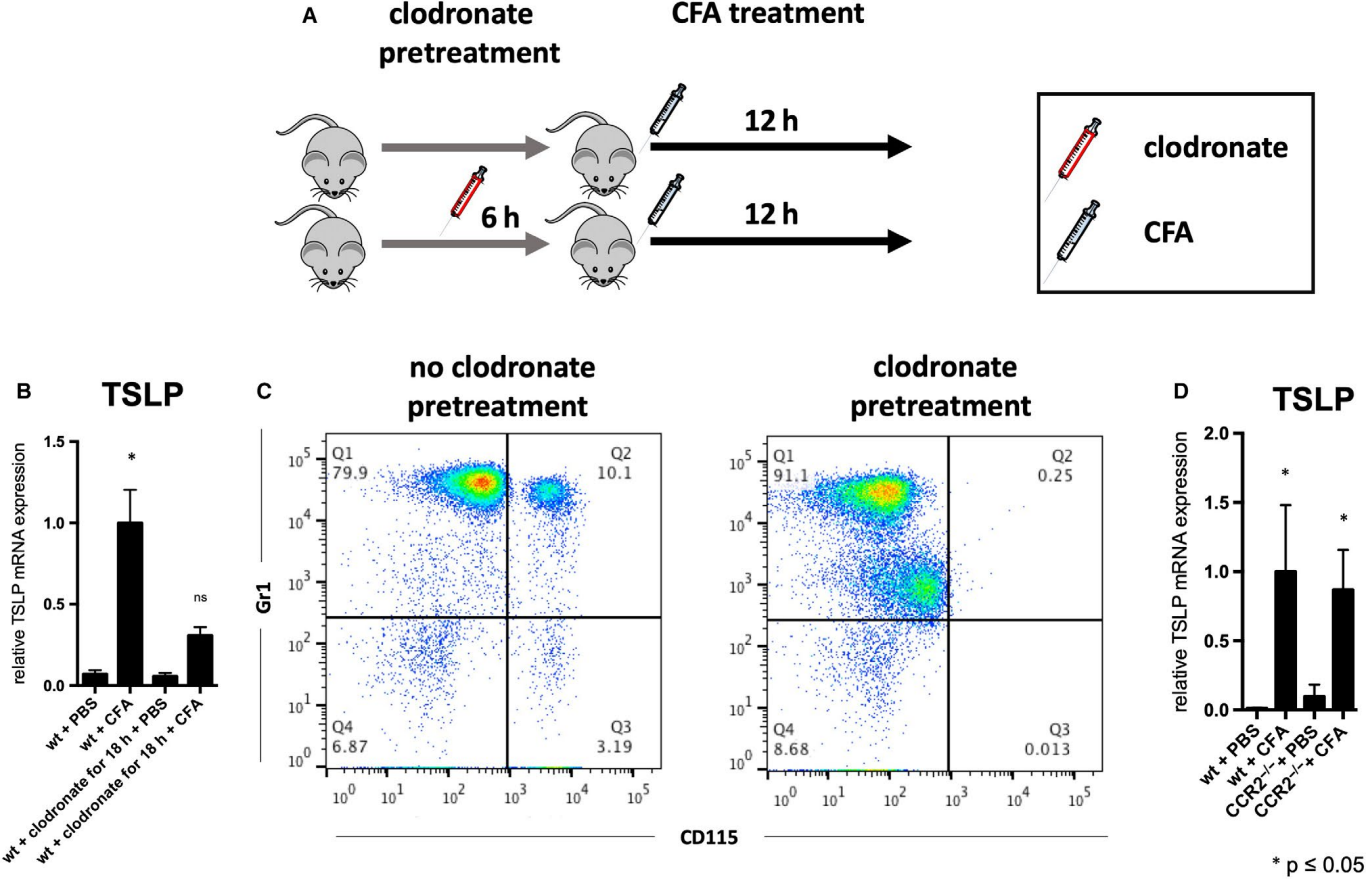
TSLP expression is altered upon monocyte depletion. A, Experimental design of clodronate depletion and CFA immunization. B, CFA‐induced TSLP mRNA expression in wild‐type mice without classical and non‐classical monocytes upon clodronate depletion (18 h). C, Flow cytometry graphs from blood samples that were retrieved at the day of killing. D, TSLP mRNA expression in the absence of classical monocytes in CCR2^−/−^ mice. *P ≤ .05 (ANOVA), n = 9‐13 for clodronate experiment, 5‐6 for CCR2^−/−^ experiment. Iv, Intravenous; wt, wild‐type mice; PBS, phosphate buffered saline; CFA, complete Freund's adjuvant

To confirm a significant role for monocytes in TSLP production, we depleted them with intravenous clodronate, and then subcutaneously injected CFA or PBS. CFA‐induced TSLP expression was significantly reduced in the absence of both classical and non‐classical monocytes (Figure 4A‐C). Interestingly, however, CFA‐induced TSLP was not altered in CCR2 deficient mice (Figure 4D), suggesting a dominant role for non‐classical monocytes in TSLP production.

### 3.4 TSLP signalling modulates type 2 immune responses upon immunization with Freund's adjuvant in males and females

**FIGURE 5:**
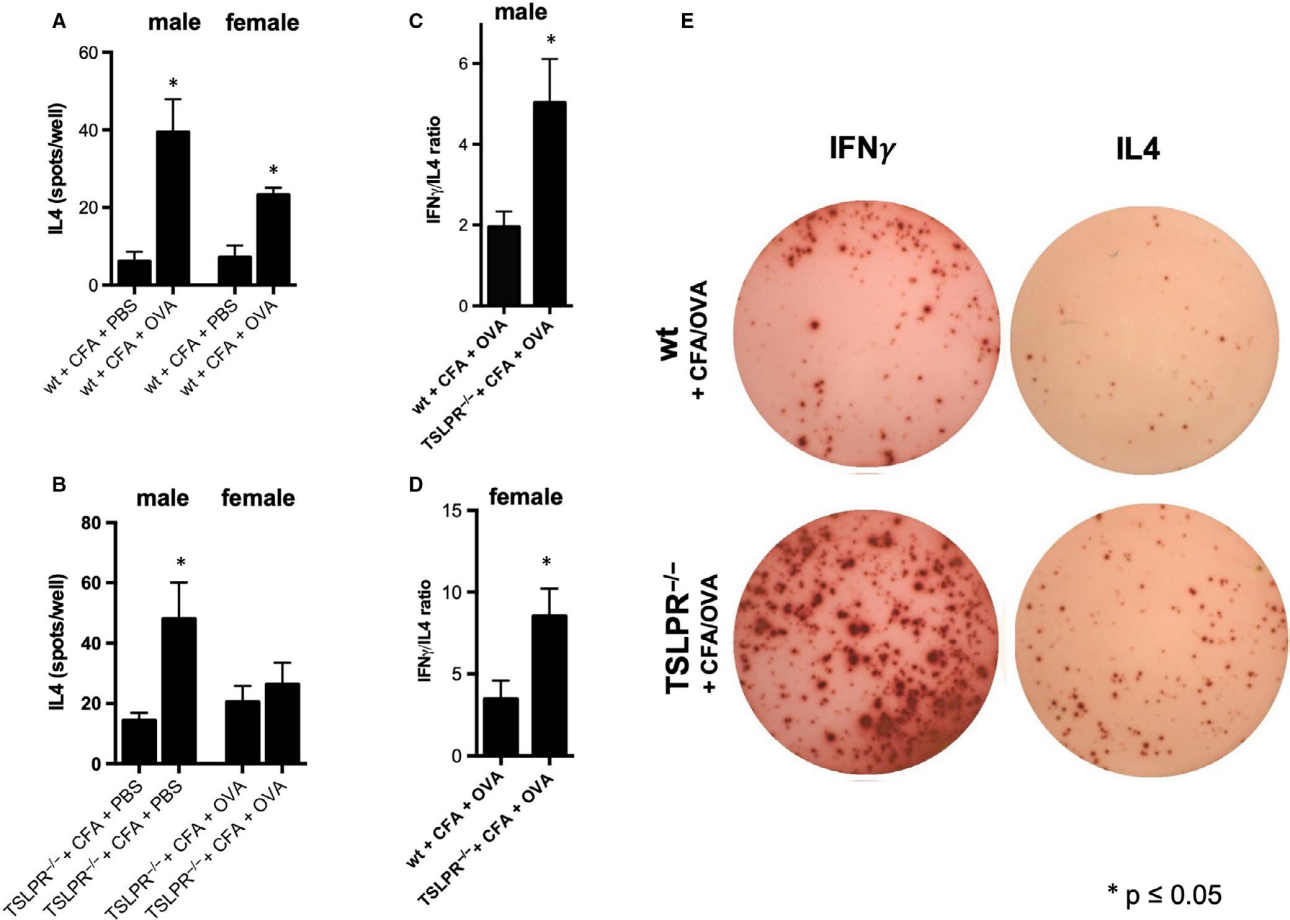
Immunization with CFA and ovalbumin yields a stronger IFNγ and attenuated IL4 response in TSLPR^−/−^ mice. A, IL4 production of splenocytes upon antigen recall 2 wk after immunization with CFA and OVA or PBS in male or female wild‐type mice (measured with ELISpot). B, IL4 production of splenocytes upon antigen recall 2 wk after immunization with CFA and OVA or PBS in male or female TSLPR^−/−^ mice (ELISpot). C, IFN*γ*/IL4 ratio upon antigen recall in male and D) female TSLPR^−/−^ mice. E) Elispot sample figures for IFNγ and IL4 secretion (ELISpot). *P ≤ .05, n = 4‐5, except for C) n = 9‐10. wt, Wild‐type mice; PBS, phosphate buffered saline; CFA, complete Freund's adjuvant; OVA, chicken ovalbumin; IL4, interleukin 4; IFN*γ*, interferon *γ*

The predominant type of inflammation in atherosclerosis features an INFγ driven, monocyte/macrophages type 1 inflammatory phenotype. Immunization models in atherosclerosis are associated with a change of this polarization and skewing towards a stronger type 2 immune response. Thus, we examined whether TSLP is relevant for the type of the immune response upon immunization with chicken Ovalbumin emulsified in CFA, and an in vitro recall 2 weeks later. Male and female wild‐type mice almost equally increase IL4 production by splenocytes upon OVA recall (Figure 5A). Interestingly, IL4 production is still increased in male TSLPR^−/−^ mice, whereas female knockout mice did not significantly respond to the antigen challenge in vitro (Figure 5B). To understand, whether TSLP‐TSLPR signalling is crucial for the immunization response to CFA/OVA not only in female but also in male mice, we repeated the experiment and analysed the response for IL4 and IFNγ. There we found, that—despite an absolute increase of both IFNγ and IL4 in male TSLPR^−/−^ mice—the INFγ/IL4 ratio (displaying single, IL4 or IFNγ producing cells) displayed a comparatively lower IL4 production in TSLPR^−/−^ mice (Figure 5C‐E). This was further supported by the OVA‐specific IgG2c/IgG1 ratio, which also suggested a skewing of the immune response towards a proportionally less Th2 and more INFγ Th1 type immunity in male TSLPR^−/−^ mice compared with wt (Figure 5F).

### 3.5 TSLP/TSLPR signalling is required for the anti‐atherogenic effect of Freund's adjuvant in ApoE^−/−^ mice

Finally, we tested whether TSLP is important for the anti‐atherosclerotic effects of Freund's adjuvant. The treatment protocol consisted of one initial CFA injection at 6 weeks of age, and 4 IFA booster immunizations at 2, 3, 4 and 5 months of age. All mice were killed at 6 months.

**FIGURE 6:**
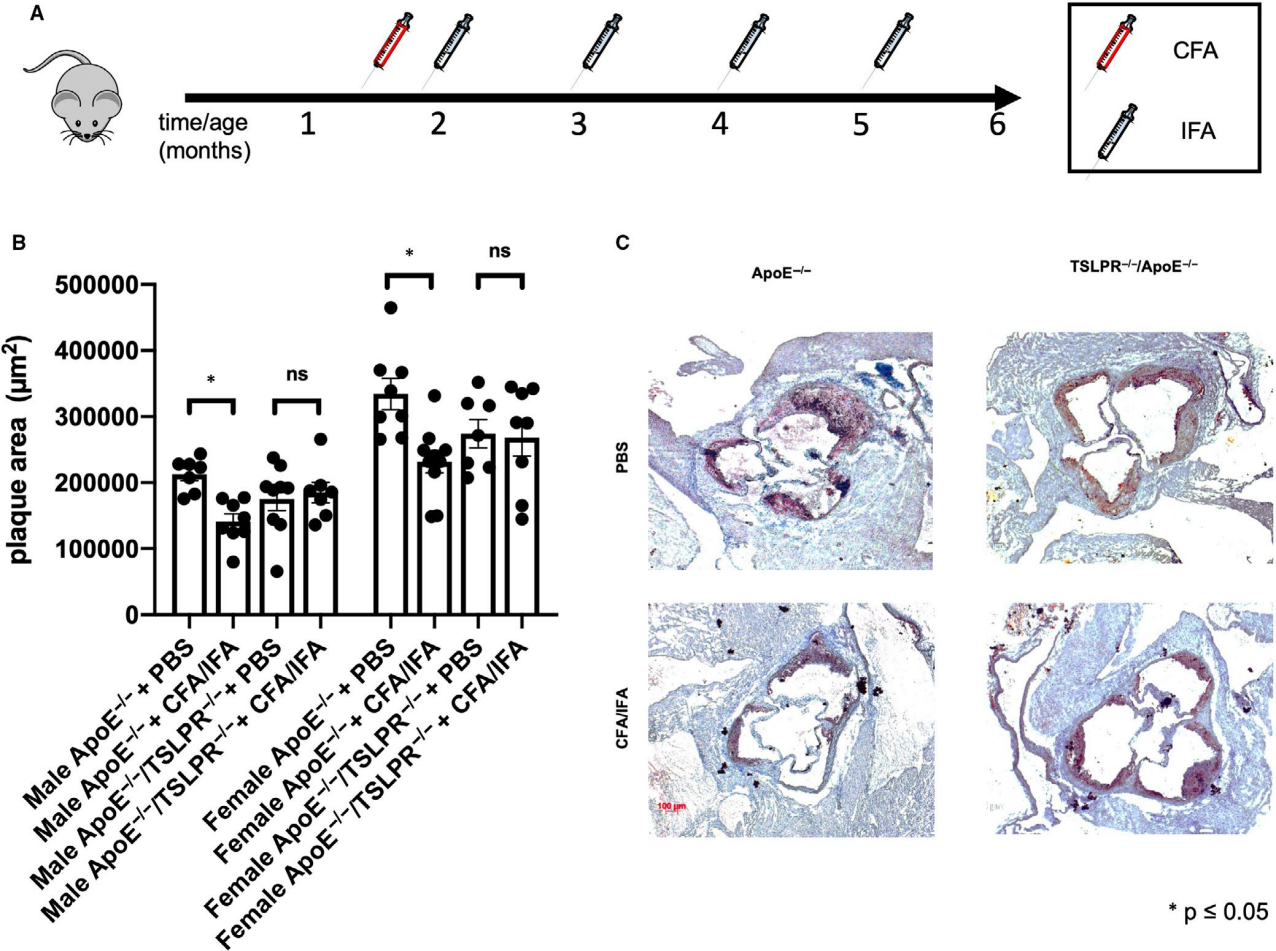
Immunization with CFA/IFA reduces atherogenesis in ApoE^−/−^ but not ApoE^−/−^/TSLPR^−/−^ mice. Male and female ApoE^−/−^ and ApoE^−/−^/TSLPR^−/−^ mice were injected CFA at 6 wk of age and then IFA at 2, 3, 4 and 5 mo of age. A, Experimental setting of immunization experiment. B, Mean aortic root plaque volume in male and female ApoE^−/−^ or ApoE^−/−^/TSLPR^−/−^ mice with indicated treatment (PBS or CFA/4xIFA). C, Examples of aortic root plaques in ApoE^−/−^ or ApoE^−/−^/TSLPR^−/−^ mice. *P ≤ .05, n = 7‐10. ApoE, Apolipoprotein E; PBS, phosphate buffered saline; CFA, complete Freund's adjuvant; IFA, incomplete Freund's adjuvant; ORO, Oil Red O; MOMA, anti‐monocyte and macrophage antibody

ApoE^−/−^ mice had significantly less plaques upon CFA/IFA treatment, whereas ApoE^−/−^/TSLPR^−/−^ mice were resistant to the atheroprotective effect of CFA/IFA treatment and featured an equal plaque burden compared with PBS controls (Figure 6). We found this result consistently in male and female ApoE^−/−^ mice, with expectedly bigger plaque burden in females (Figure 6).

## 4 DISCUSSION

Directing inflammation in order to benefit atherosclerosis is a promising approach with high potential for translation from mice to humans. In mouse models, treatment with some immune adjuvants restrains the development of atherosclerotic plaques,^7^ although the mechanisms behind this protection have not been addressed in detail. Given previous indications that atheroprotection in response to Alum and Freund's adjuvants was associated with a shift towards a type 2 immune response,^14^, ^15^ we set out to address the underlying mechanisms.

In our study, we show that Freund's and Alum, known atheroprotective adjuvants, induce the expression of TSLP at the injection site. Interestingly, however, TSLP expression is not affected by CpG, an adjuvant that does not alter the development of atherosclerosis. Thus, there was a correlation between the atheroprotective properties of adjuvants and their ability to induce TSLP expression in skin after subcutaneous injection. We further focused on Freund's adjuvant to dissect the mechanisms behind adjuvant‐induced TSLP. The mechanisms of CFA‐induced TSLP differ in males and females. While the mechanisms responsible for TSLP induction in females are still poorly understood, CFA‐induced TSLP requires NLRP3 inflammasome‐dependent IL1β production in males. Although Alum particulates have been reported to activate NLRP3 inflammasome,^16^ the impact of Freund's adjuvant on NLRP3 expression or activation may differ.^16^, ^17^ Importantly, the mycobacterial component in CFA is not needed for the expression of TSLP, since IFA has the same effects in our model. This is also consistent with the fact that MINCLE, the receptor for mycobacterial cord factor was not required for CFA‐induced TSLP (Figure S2).

Based on the study by Tang et al who found epidermis‐derived TSLP was pivotal for the papain effect on T‐cell polarization,^12^ we initially hypothesized that mouse keratinocytes might be the major source for TSLP upon CFA injection. This possibility was reliably excluded using immunohistochemistry, which showed TSLP expression in MOMA+ cells, not keratinocytes, suggesting a monocyte/macrophage source. We also detected TSLP in CD11b‐positive cells from digested skin samples that were injected with CFA. Furthermore, monocyte depletion experiments and use of CCR2 deficient mice strongly suggested a role for non‐classical monocytes in TSLP expression in our model. Whereas the induction of TSLP by IL1β is best established in human and murine keratinocytes in atopic dermatitis,^18^, ^19^, ^20^ other cell types including immune cells may substantially contribute to TSLP production. For example, IL1β positively modulates TSLP production and secretion in DCs in vitro,^21^ and in a recent study, myeloid cells, including neutrophils and monocytes, have been shown to produce TSLP in response to IL1α, a mechanism that was responsible for breast cancer spreading.^22^

When we tested the relevance of TSLP/TSLPR signalling in immunization with CFA through antigen exposure, we found that TSLP was crucial to balance IFN*γ* and IL4 production. TSLPR deficiency in females abrogated IL4 production upon (re)exposition of total splenocytes to OVA, and even though IL4 was still produced to some extent in the absence of TSLPR in males, IFN*γ* production was dramatically boosted leading to a significant increase of IFN*γ*/IL4 ratio, and a likewise increase of IgG2c/IgG1 ratio. Thus, our data identify a critical role for TSLP in the modulation of the immune response to Freund's adjuvant. The role of TSLP in promoting a type 2 immune response is consistent with previous observations. TSLP production by DCs fosters a Th2 polarization when interacting with T cells,^23^ and TSLP has been shown responsible for the Th2 polarizing capacity of cysteine‐protease papain.^12^ TSLP has been used itself as an mucosal immuno‐adjuvant by van Roey and colleagues, who showed a significant on inflammatory response skewing towards a Th2 cytokine profile.^24^ TSLP, together with IL25 or IL33, is a regulator of the proliferation and maturation of group 2 innate lymphoid cells (ILC2),^25^ which are important for Th2 responses orchestrated in the skin.

Finally, our study establishes a critical role for TSLPR signalling in CFA/IFA‐mediated inhibition of atherogenesis in ApoE^−/−^ mice. Recent studies have addressed the role of TSLP in murine atherosclerosis with partly conflicting results. Wu et al hypothesized a pro‐atherosclerotic role for TSLP, because ApoE^−/−^TSLPR^−/−^ mice on a high fat diet had less atherosclerosis than ApoE^−/−^ controls.^26^ Another group described a strong expression of TSLP in human atherosclerotic plaques.^27^ On the contrary, Yu and co‐workers reported an attenuation of atherosclerosis following TSLP administration in mice.^28^ Our data support a protective role of TSLP/TSLPR signalling in a specific setting of induction of type 2 immune responses. Importantly, we investigated atherogenesis in a model with only mild hypercholesterolaemia, in which both innate and adaptive immune responses significantly contribute to atherogenesis. Of note, ApoE^−/−^ mice fed a high fat and cholesterol rich diet as used by Wu et al, display very severe hypercholesterolaemia and exaggerated innate immune responses,^2^ but importantly a negligible role for adaptive immune cells, that is T and B lymphocytes.^29^ This may provide some explanation for the discrepant findings under different diet conditions.

In summary, we have shown that TSLP is critically involved in the induction of type 2 immune responses to Freund's adjuvant. In males, this occurs through NLRP3 inflammasome and IL1*β*‐dependent expression of TSLP. TSLP/TSLPR signalling also mediates the atheroprotective effect of Freund's adjuvant in ApoE^−/−^ mice.

## CONFLICT OF INTEREST

The authors have no conflicts of interest.

## AUTHOR CONTRIBUTIONS

MS designed and performed experiments, analysed data, wrote article; LL, SN, LW, BV, PP, BE, MV, AG, CG, ES, TR and SP performed experiments; GN and TR contributed materials and analysis tools; AT conceived and designed experiments, analysed data; Z conceived and designed experiments, contributed materials and analysis tools, analysed data and wrote article; all authors read and approved the final manuscript.

## Supporting information

Fig S1

Fig S2

Fig S3

Fig S4

Fig S5

Fig S6

Table S1

Supplementary Material

## Data Availability

The data that support the findings of this study included in the study or available from the corresponding author, MS, upon reasonable request.
