# Supplementary figures and images for: Thymic stromal lymphopoietin is a key cytokine for the immunomodulation of atherogenesis with Freund's adjuvant
Source: J Cell Mol Med. 2020 Apr 13;24(10):5731–9. doi: 10.1111/jcmm.15235 (PMC7214169; doi:10.1111/jcmm.15235)

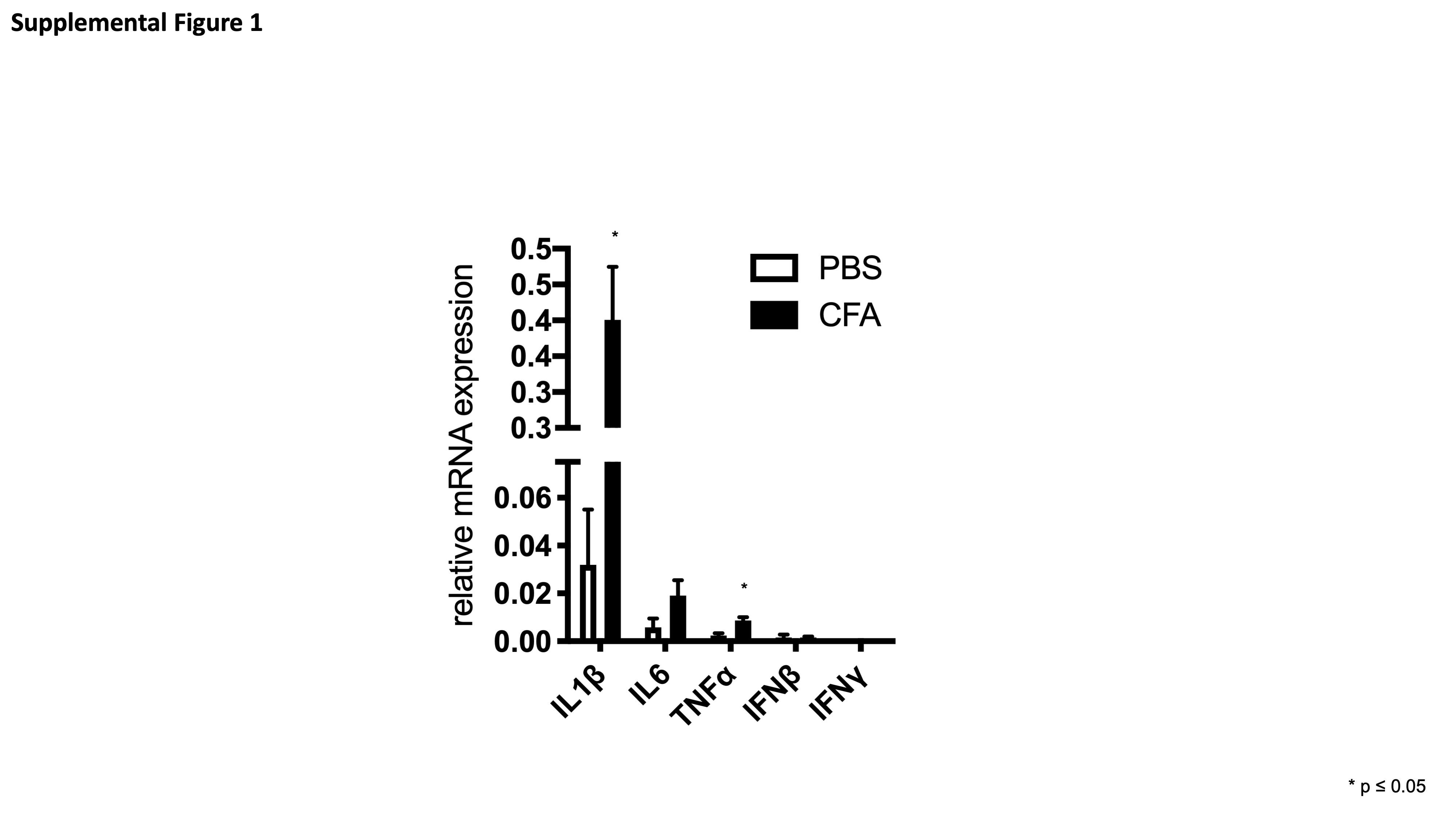

Supplement: Supplementary file 1 — Fig S1 [file JCMM-24-5731-s001.jpeg]

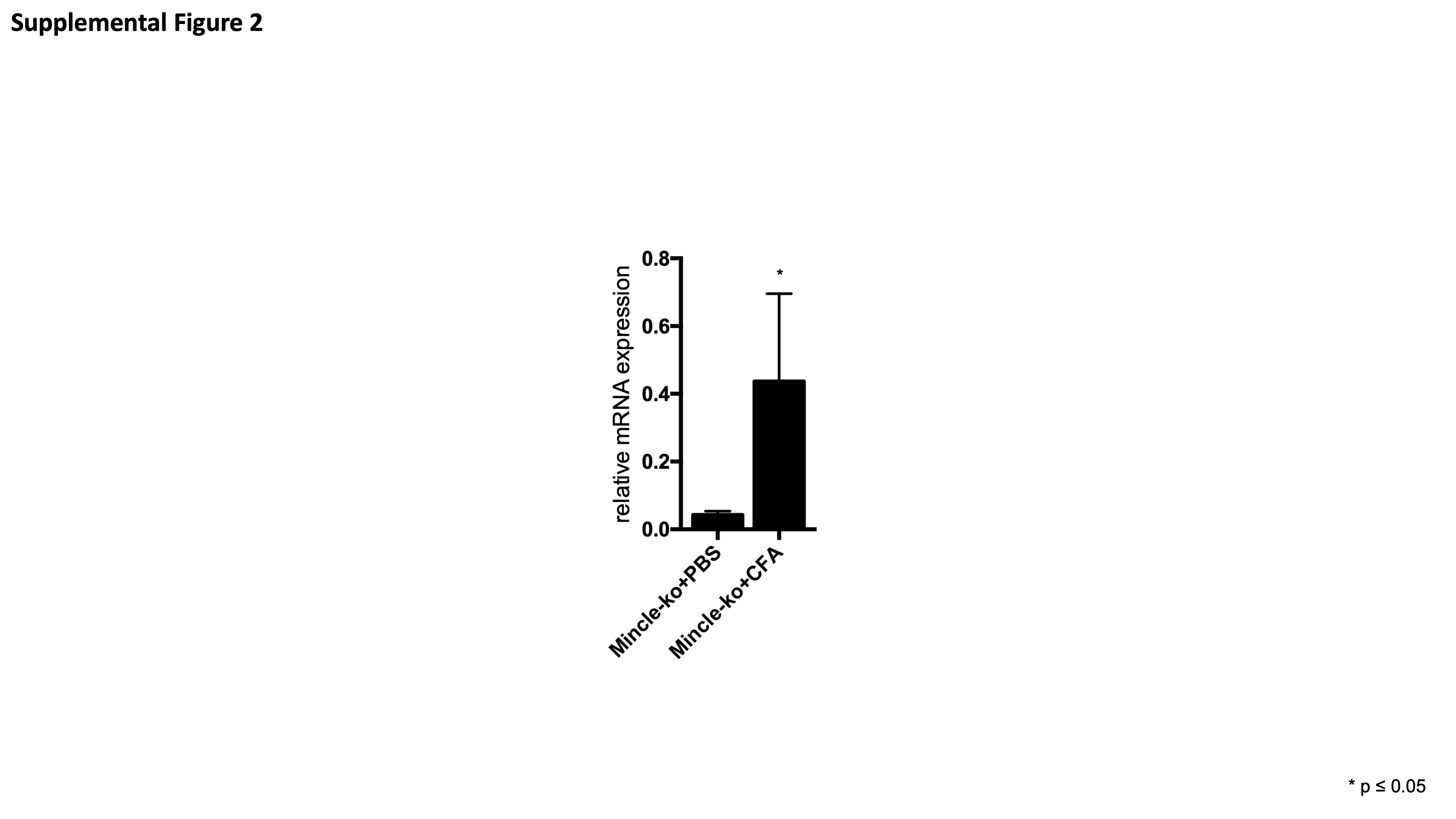

Supplement: Supplementary file 2 — Fig S2 [file JCMM-24-5731-s002.jpeg]

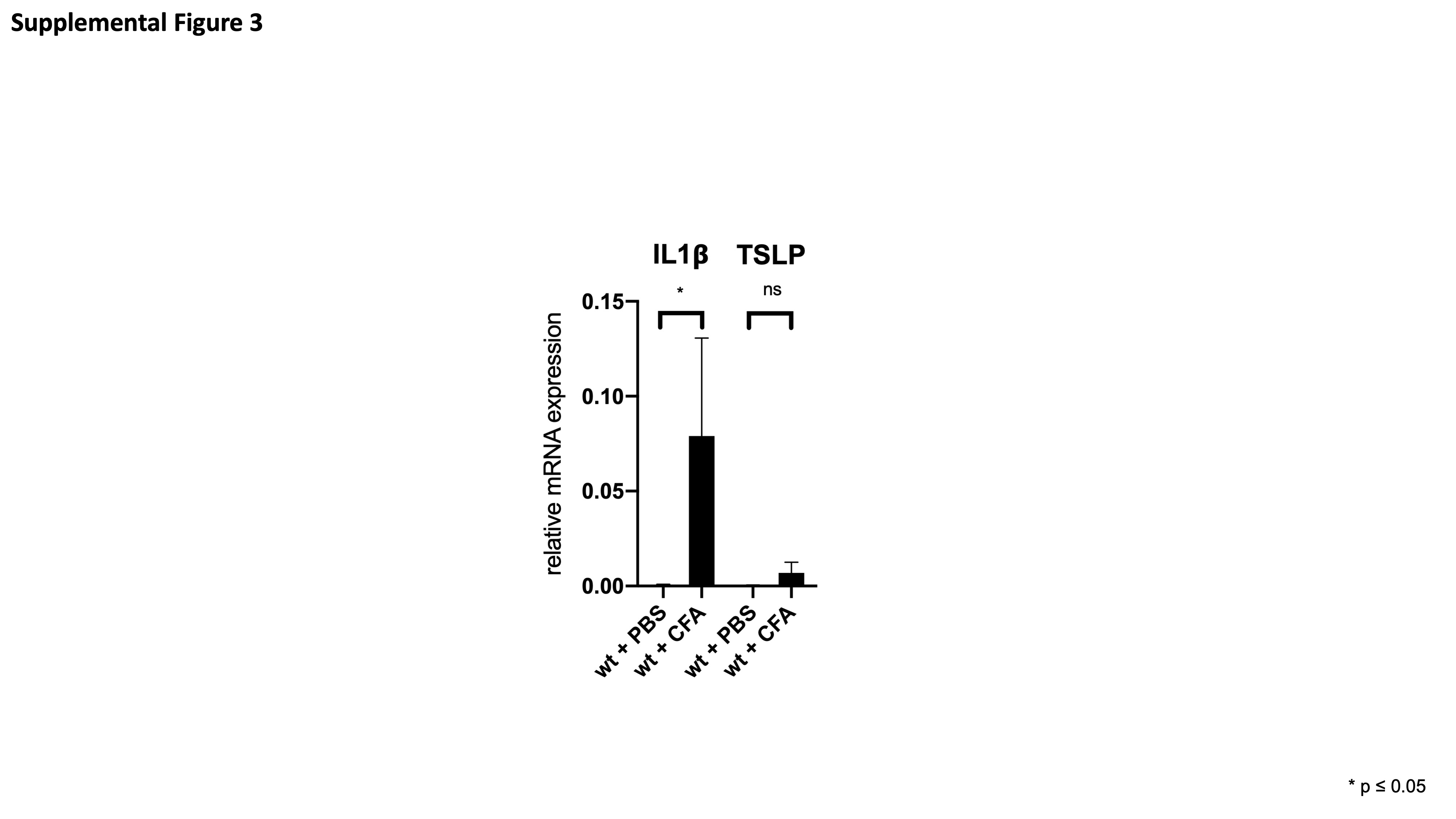

Supplement: Supplementary file 3 — Fig S3 [file JCMM-24-5731-s003.jpeg]

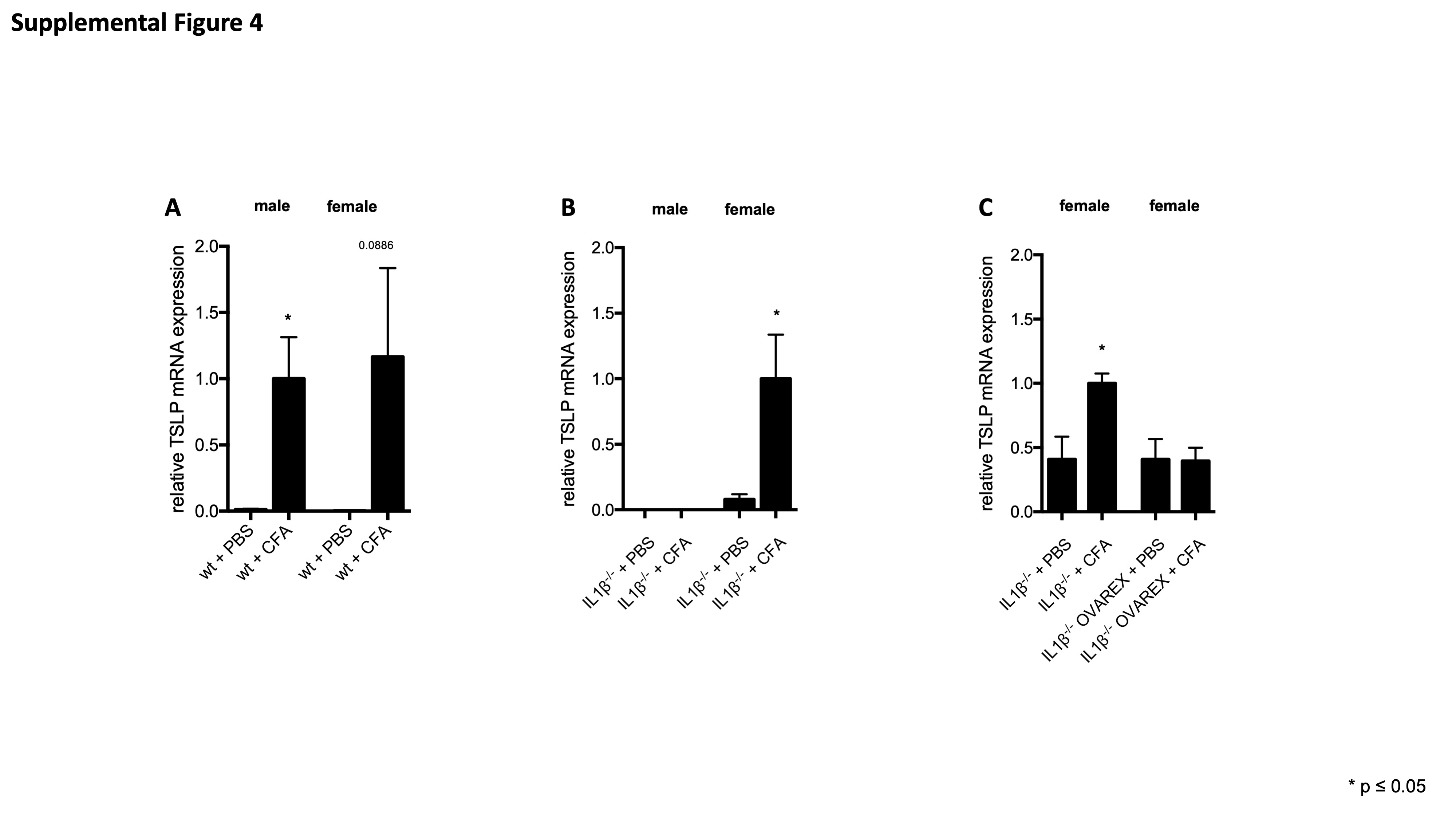

Supplement: Supplementary file 4 — Fig S4 [file JCMM-24-5731-s004.jpeg]

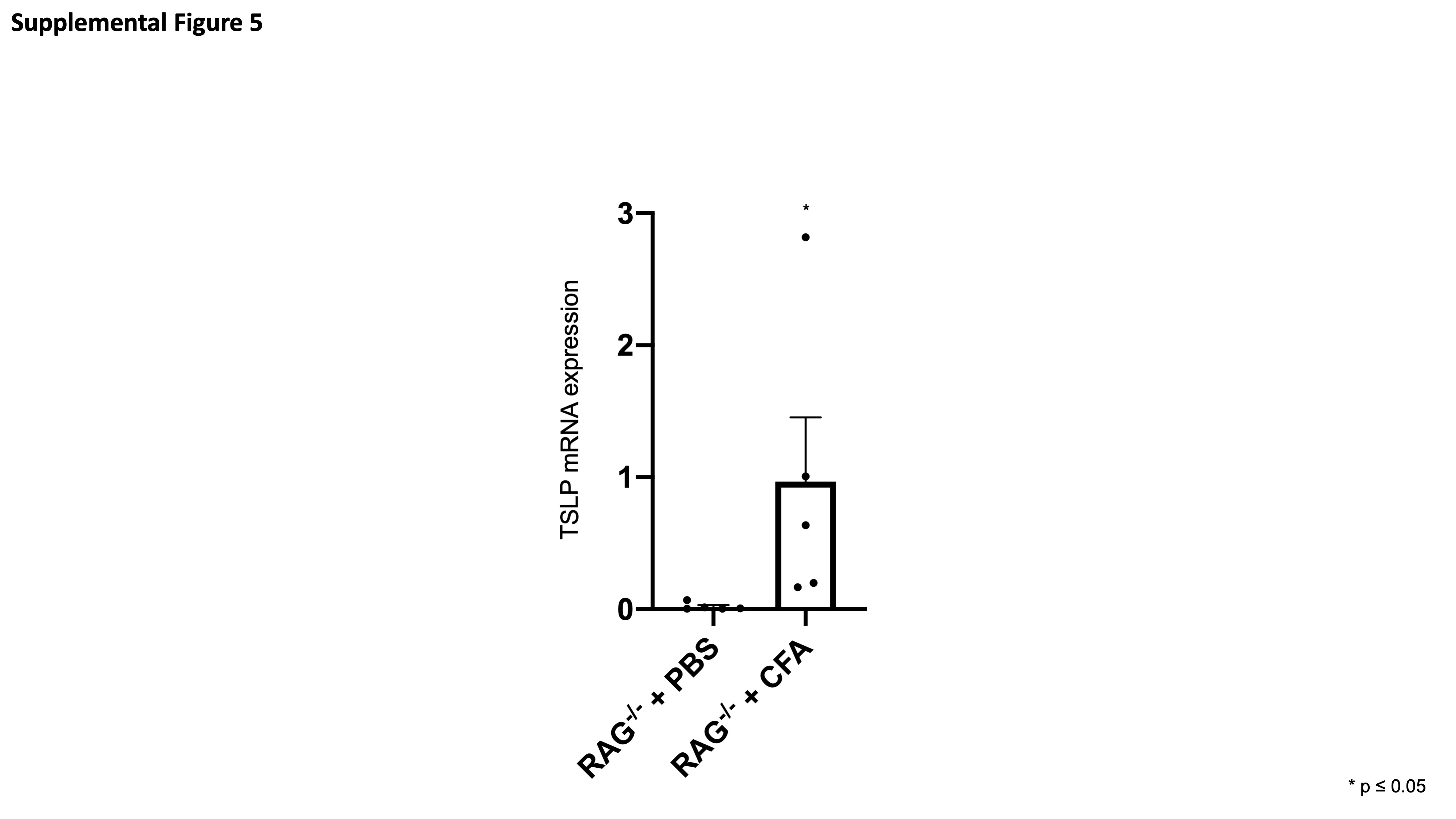

Supplement: Supplementary file 5 — Fig S5 [file JCMM-24-5731-s005.jpeg]

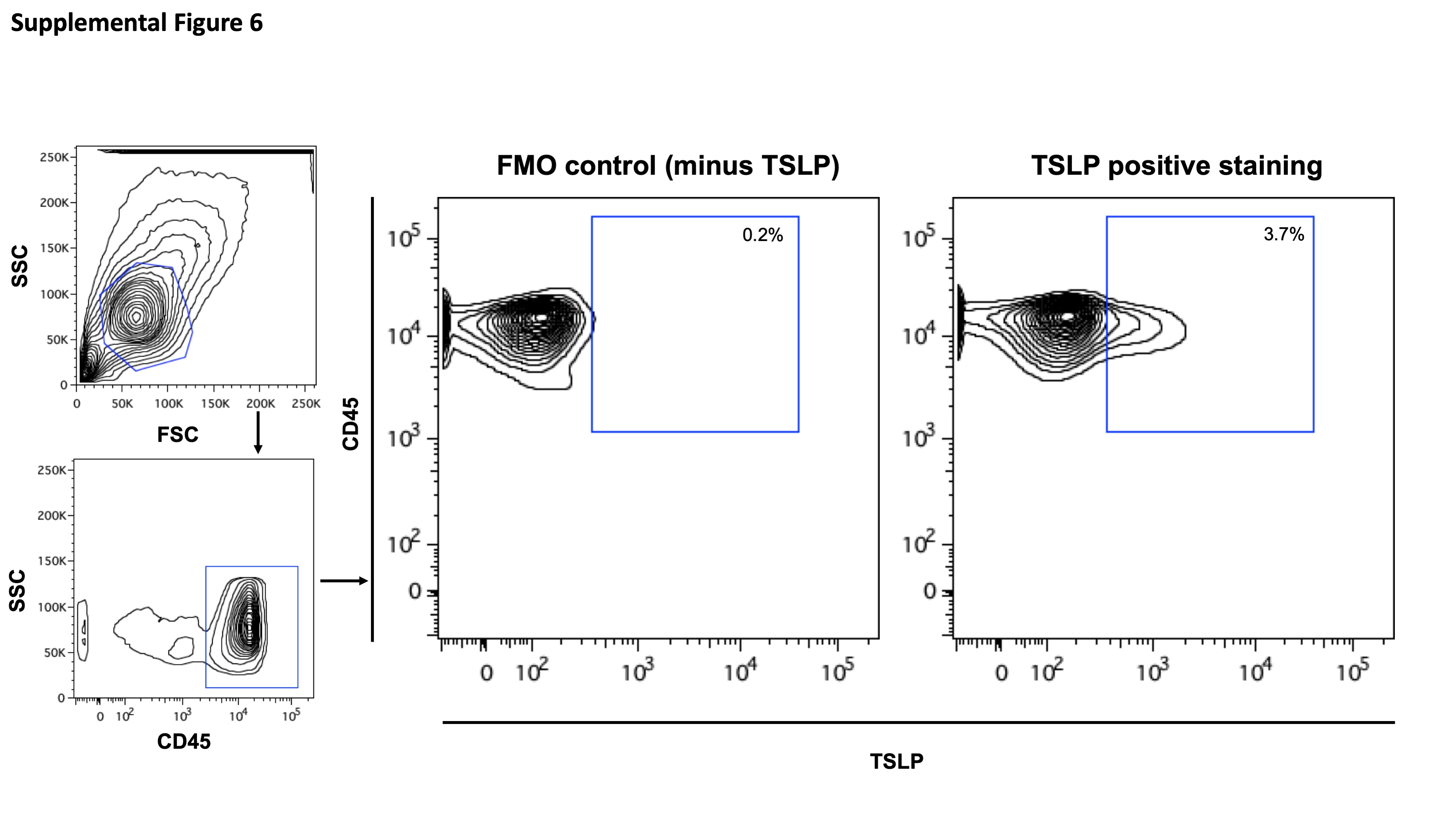

Supplement: Supplementary file 6 — Fig S6 [file JCMM-24-5731-s006.jpeg]
