# Supplementary material for: Thymic stromal lymphopoietin is a key cytokine for the immunomodulation of atherogenesis with Freund's adjuvant
Source: J Cell Mol Med. 2020 Apr 13;24(10):5731–9. doi: 10.1111/jcmm.15235 (PMC7214169; doi:10.1111/jcmm.15235)
Supplement: Supplementary file 7 — Table S1 [file JCMM-24-5731-s007.docx]

| **Related figure** | **Mouse group** | **Treatment regimen** | **Type of injection** | **Duration of immunization before sacrifice** | **Type of mouse** | **Annotation** |
| --- | --- | --- | --- | --- | --- | --- |
| 1A | PBS | PBS | s.c. | 12 hours | C57BL/6 |  |
|  | papain | papain | s.c. | 12 hours | C57BL/6 |  |
|  | CFA | CFA | s.c. | 12 hours | C57BL/6 |  |
|  | oxLDL | oxLDL | s.c. | 12 hours | C57BL/6 |  |
|  | MDA-LDL | MDA-LDL | s.c. | 12 hours | C57BL/6 |  |
| 1B | PBS | PBS | s.c. | 12 hours | C57BL/6 |  |
|  | CFA | CFA | s.c. | 12 hours | C57BL/6 |  |
|  | IFA | IFA | s.c. | 12 hours | C57BL/6 |  |
|  | Alum | Alum | s.c. | 12 hours | C57BL/6 |  |
|  | CpG | CpG | s.c. | 12 hours | C57BL/6 |  |
|  | scramble | Scramble control to CpG | s.c. | 12 hours | C57BL/6 |  |
| 1C | control | PBS | s.c. | 12 hours | C57BL/6 |  |
|  | 12h | CFA or Alum (as indicated) | s.c. | 12 hours | C57BL/6 |  |
|  | 24h | CFA or Alum (as indicated) | s.c. | 24 hours | C57BL/6 |  |
|  | 72h | CFA or Alum (as indicated) | s.c. | 72 hours | C57BL/6 |  |
| 1D | control | PBS | s.c. | 12 hours | C57BL/6 |  |
|  | 12h | CFA or Alum (as indicated) | s.c. | 12 hours | C57BL/6 |  |
|  | 24h | CFA or Alum (as indicated) | s.c. | 24 hours | C57BL/6 |  |
|  | 72h | CFA or Alum (as indicated) | s.c. | 72 hours | C57BL/6 |  |
| 1E | wt + PBS | PBS | s.c. | 12 hours | C57BL/6 |  |
|  | wt + CFA | CFA | s.c. | 12 hours | C57BL/6 |  |
|  | ApoE^-/-^ + PBS | PBS | s.c. | 12 hours | ApoE^-/-^ |  |
|  | ApoE^-/-^ + CFA | CFA | s.c. | 12 hours | ApoE^-/-^ |  |
| 2A | wt + PBS | PBS | s.c. | 12 hours | C57BL/6 |  |
|  | wt + CFA | CFA | s.c. | 12 hours | C57BL/6 |  |
|  | IL1𝛽^-/-^ + PBS | PBS | s.c. | 12 hours | IL1𝛽^-/-^ |  |
|  | IL1𝛽^-/-^ + CFA | CFA | s.c. | 12 hours | IL1𝛽^-/-^ |  |
| 2B | wt + PBS | PBS | s.c. | 12 hours | C57BL/6 |  |
|  | wt + CFA | CFA | s.c. | 12 hours | C57BL/6 |  |
|  | TSLPR^-/-^ + PBS | PBS | s.c. | 12 hours | TSLPR^-/-^ |  |
|  | TSLPR^-/-^ + CFA | CFA | s.c. | 12 hours | TSLPR^-/-^ |  |
| 2C | wt + PBS | PBS | s.c. | 12 hours | C57BL/6 |  |
|  | wt + CFA | CFA | s.c. | 12 hours | C57BL/6 |  |
|  | NLRP3^-/-^ + PBS | PBS | s.c. | 12 hours | NLRP3^-/-^ |  |
|  | NLRP3^-/-^ + CFA | CFA | s.c. | 12 hours | NLRP3^-/-^ |  |
|  |  |  |  |  |  |  |
| 3A-C | wt + PBS | PBS | s.c. | 12 hours | C57BL/6 |  |
|  | wt + CFA | CFA | s.c. | 12 hours | C57BL/6 |  |
|  |  |  |  |  |  |  |
| 4B | wt + PBS | PBS | s.c. | 12 hours | C57BL/6 |  |
|  | wt + CFA | CFA | s.c. | 12 hours | C57BL/6 |  |
|  | wt + clodronate for 18 hours + PBS | Clodronate, PBS | i.v.,  s.c. | Clodronate 18 hours (6 hours prior to PBS injection);  PBS: 12 hours | C57BL/6 |  |
|  | wt + clodronate for 18 hours + CFA | Clodronate,  PBS | i.v.,  s.c. | Clodronate 18 hours (6 hours prior to CFA injection);  CFA: 12 hours | C57BL/6 |  |
| 5A | male wt + CFA + PBS | CFA+PBS (i.e. no antigen) | s.c. | 2 weeks | C57BL/6 | in vitro recall with OVA |
|  | male wt + CFA + OVA | CFA + OVA | s.c. | 2 weeks | C57BL/6 | in vitro recall with OVA |
|  | female wt + CFA + PBS | CFA+PBS (i.e. no antigen) | s.c. | 2 weeks | C57BL/6 | in vitro recall with OVA |
|  | female wt + CFA + OVA | CFA + OVA | s.c. | 2 weeks | C57BL/6 | in vitro recall with OVA |
| 5B | male TSLPR^-/-^ + CFA + PBS | CFA+PBS (i.e. no antigen) | s.c. | 2 weeks | TSLPR^-/-^ | in vitro recall with OVA |
|  | male TSLPR^-/-^ + CFA + OVA | CFA + OVA | s.c. | 2 weeks | TSLPR^-/-^ | in vitro recall with OVA |
|  | female TSLPR^-/-^ + CFA + PBS | CFA+PBS (i.e. no antigen) | s.c. | 2 weeks | TSLPR^-/-^ | in vitro recall with OVA |
|  | female TSLPR^-/-^ + CFA + OVA | CFA + OVA | s.c. | 2 weeks | TSLPR^-/-^ | in vitro recall with OVA |
| 5C | male wt + CFA + OVA | CFA + OVA | s.c. | 2 weeks | C57BL/6 | in vitro recall with OVA |
|  | male TSLPR^-/-^ + CFA + OVA | CFA + OVA | s.c. | 2 weeks | TSLPR^-/-^ | in vitro recall with OVA |
| 5D | female TSLPR^-/-^ + CFA + OVA | CFA + OVA | s.c. | 2 weeks | TSLPR^-/-^ | in vitro recall with OVA |
|  | female TSLPR^-/-^ + CFA + OVA | CFA + OVA | s.c. | 2 weeks | TSLPR^-/-^ | in vitro recall with OVA |
| 6B | male ApoE^-/-^ + PBS | 5xPBS | s.c. | 4,5 months  (at age 6 months) | ApoE^-/-^ |  |
|  | male ApoE^-/-^ + CFA/IFA | CFA/4xIFA | s.c. | 4,5 months  (at age 6 months) | ApoE^-/-^ |  |
|  | male ApoE^-/-^/TSLPR^-/-^ + PBS | 5xPBS | s.c. | 4,5 months  (at age 6 months) | ApoE^-/-^/TSLPR^-/-^ |  |
|  | male ApoE^-/-^/TSLPR^-/-^ + CFA/IFA | CFA/4xIFA | s.c. | 4,5 months  (at age 6 months) | ApoE^-/-^/TSLPR^-/-^ |  |
|  | female ApoE^-/-^ + PBS | 5xPBS | s.c. | 4,5 months  (at age 6 months) | ApoE^-/-^ |  |
|  | female ApoE^-/-^ + CFA/IFA | CFA/4xIFA | s.c. | 4,5 months  (at age 6 months) | ApoE^-/-^ |  |
|  | female ApoE^-/-^/TSLPR^-/-^ + PBS | 5xPBS | s.c. | 4,5 months  (at age 6 months) | ApoE^-/-^/TSLPR^-/-^ |  |
|  | female ApoE^-/-^/TSLPR^-/-^ + CFA/IFA | CFA/4xIFA | s.c. | 4,5 months  (at age 6 months) | ApoE^-/-^/TSLPR^-/-^ |  |
|  |  |  |  |  |  |  |
| **Suppl. figures** |  |  |  |  |  |  |
| S1 | wt + PBS | PBS | s.c. | 12 hours | C57BL/6 |  |
|  | wt + CFA | CFA | s.c. | 12 hours | C57BL/6 |  |
| S2 | Mincle-ko + PBS | PBS | s.c. | 12 hours | CLEC4E^-/-^ |  |
|  | Mincle-ko + CFA | CFA | s.c. | 12 hours | CLEC4E^-/-^ |  |
| S3 | wt + PBS | PBS | s.c. | 12 hours | C57BL/6 |  |
|  | wt + CFA | CFA | s.c. | 12 hours | C57BL/6 |  |
| S4A | male wt + PBS | PBS | s.c. | 12 hours | C57BL/6 |  |
|  | male wt + CFA | CFA | s.c. | 12 hours | C57BL/6 |  |
|  | female wt + PBS | PBS | s.c. | 12 hours | C57BL/6 |  |
|  | female wt + CFA | CFA | s.c. | 12 hours | C57BL/6 |  |
| S4B | male IL1𝛽^-/-^ + PBS | PBS | s.c. | 12 hours | IL1𝛽^-/-^ |  |
|  | male IL1𝛽^-/-^ + CFA | CFA | s.c. | 12 hours | IL1𝛽^-/-^ |  |
|  | female IL1𝛽^-/-^ + PBS | PBS | s.c. | 12 hours | IL1𝛽^-/-^ |  |
|  | female IL1𝛽^-/-^ + CFA | CFA | s.c. | 12 hours | IL1𝛽^-/-^ |  |
| S4C | female IL1𝛽^-/-^ + PBS | PBS | s.c. | 12 hours | IL1𝛽^-/-^ |  |
|  | female IL1𝛽^-/-^ + CFA | CFA | s.c. | 12 hours | IL1𝛽^-/-^ |  |
|  | female IL1𝛽^-/-^ OVAREX + PBS | PBS | s.c. | 12 hours | IL1𝛽^-/-^ | ovariectomized |
|  | female IL1𝛽^-/-^ OVAREX + CFA | CFA | s.c. | 12 hours | IL1𝛽^-/-^ | ovariectomized |
| S5 | RAG^-/-^ + PBS | PBS | s.c. | 12 hours | RAG1^-/-^ |  |
|  | RAG^-/-^ + CFA | CFA | s.c. | 12 hours | RAG1^-/-^ |  |
