# Supplementary material for: Thymic stromal lymphopoietin is a key cytokine for the immunomodulation of atherogenesis with Freund's adjuvant
Source: J Cell Mol Med. 2020 Apr 13;24(10):5731–9. doi: 10.1111/jcmm.15235 (PMC7214169; doi:10.1111/jcmm.15235)
Supplement: Supplementary file 8 — Supplementary Material [file JCMM-24-5731-s008.docx]

**Supplemental Figure 1.** *Pro-inflammatory cytokines at the site of injection upon CFA s.c. administration.* * p ≤ 0.05, n=4

*Abbreviations****:*** interleukin-1𝛽 (IL1𝛽), interleukin-6 (IL6), tumour necrosis factor-𝛼 (TNF𝛼), interferon-𝛽 (IFN𝛽), interferon-𝛾 (IFN𝛾)

**Supplemental Figure 2.** *TSLP in MINCLE-deficient mice (Mincle-ko) upon s.c. CFA administration. * p ≤ 0.05, n=4-5*

*Abbreviations****:*** macrophage-inducible C-type lectin (MINCLE)

**Supplemental Figure 3.** *Pro-inflammatory cytokines upon CFA s.c. administration, time-point 4 hours after injection. * p ≤ 0.05, n=4-5; exact p values: IL1𝝱 = 0.016, TSLP 0.19*

*Abbreviations****:*** interleukin-1𝛽 (IL1𝛽)

**Supplemental Figure 4.** *TSLP induction in males and females upon s.c. CFA administration.* A) Male vs female wild-type mice ± PBS or CFA. B) Male vs female IL1𝛽*^-^*^/-^ mice ± PBS or CFA. C) Female IL1𝛽*^-^*^/-^ mice vs ovariectomized female IL1𝛽*^-^*^/-^ mice ± PBS or CFA.

* p ≤ 0.05, n=4-5

*Abbreviations*: wild-type (wt), phosphate buffered saline (PBS), complete Freund‘s adjuvant (CFA), ovarectomized (OVAREX)

**Supplemental Figure 5.** Relative TSLP mRNA expression in RAG-deficient (RAG^-^/^-^) mice at the site of injection upon treatment with PBS or CFA *.*

* p ≤ 0.05, n=5

*Abbreviations****:*** recombination activating gene (RAG)

**Supplemental Figure 6.** *Gating strategy.* FMO (fluorescence minus one, no TSLP added) control vs staining including TSLP from digested skin samples. Gating hierarchy indicated by arrows.

**Supplemental Table 1.** Overview of all animal experiments and experiment designs.
